# Supplementary material for: Novel Variants in MPV17, PRX, GJB1, and SACS Cause Charcot–Marie–Tooth and Spastic Ataxia of Charlevoix–Saguenay Type Diseases
Source: Genes (Basel). 2023 Jan 27;14(2):328. doi: 10.3390/genes14020328 (PMC9956329; doi:10.3390/genes14020328)
Supplement: Supplementary file 1 [file genes-14-00328-s001.zip › genes-2138525-supplementary.pdf]

Supplementary Table S1: The classification of different types of Charcot-Marie-Tooth (CMT) disease includes subtypes, their phenotypes, OMIM ID, mode of inheritance, responsible genes, chromosomal location, protein, UniProtKB ID, and onset age.

**Charcot-Marie-Tooth Disease Type 1 (CMT1)**									
**S/No.**	**Subtypes**	**CMT OMIM**	**Inheritanc e**	**Gene**	**Gene OMIM**	**Chromosoma l location**	**Protein**	**UniprotKB**	**Onset age**
1.1	CMT type 1A	118220	AD	*PMP22*	601097	17p12	PMP22	Q01453	1st-2nd decade
1.2	CMT type 1B	118200	AD	*MPZ*	159440	1q23.3	MPZ	P25189	1st -2nd decade
1.3	CMT type 1C	601098	AD	*LITAF*	603795	16p13.13	LITAF	Q99732	2-12 years
1.4	CMT type 1D	607678	AD	*EGR2*	129010	10q21.3	EGR2	P11161	1st-2nd decade
1.5	CMT type 1E	118300	AD	*PMP22*	601097	17p12	PMP22	Q01453	2-12 years
1.6	CMT type 1F	607734	AD/AR	*NEFL*	162280	8p21.2	NEFL	P07196	1-13 years
1.7	CMT type1G	6168279	AD	*PMP2*	170715	8q21.13	PMP2	P02689	1st -2nd decade
1.8	CMT type1H	619764	AD	*FBLN5*	604580	14q32.12	FBLN5	Q9UBX5	1st- 3rd Decade
1.9	CMT type1I	619742	AD	*POLR3B*	614366	12q23.3	POLR3B	Q9NW08	1st -2nd decade
**Charcot-Marie-Tooth Disease Type 2 (CMT2)**									
2.1	CMT type 2A1	118210	AD	*KIF1B*	605995	1p36.22	KIF1B	O60333	1st – 5th decade
2.2	CMT type 2A2	609260	AD/AR	*MFN2*	608507	1p36.22	MFN2	O95140	1st – 5th decade
2.3	CMT type 2B	600882	AD	*RAB7*	602298	3q21.3	RAB7	P51149	2nd Decade
2.3.1	CMT type 2B1	605588	AR	*LMNA*	150330	1q22	LMNA	P02545	1st- 3rd Decade
2.3.2	CMT type 2B2	605589	AR	*MED25*	605610	19q13.33	MED25	Q71SY5	3rd – 5th decade

2.4	CMT type 2C	606071	AD	*TRPV4*	605427	12q24.11	TRPV4	Q9HBA0	1st – 6th decade
2.5	CMT type 2CC	616924	AD	*NEFH*	162230	22q12.2	NEFH	P12036	1st – 4th Decade
2.6	CMT type 2D	601472	AD	*GARS1*	600287	7p14.3	GARS1	P41250	2nd –3rd decade
2.7	CMT type 2DD	618036	AD	*ATP1A1*	182310	1p13.1	ATP1A1	P05023	1st -2nd decade
2.8	CMT type 2E	607684	AD	*NEFM*	162280	8p21.2	NEFM	P07197	1st- 3rd Decade
2.9	CMT type 2EE	618400	AR	*MPV17*	137960	2p23.3	MPV17	P39210	1st -2nd Decade
2.10	CMT type 2F	606595	AD	*HSPB1*	602195	7q11.23	HSPB1	P04792	2nd –3rd decade
2.11	CMT type 2FF	619519	AD	*CADM3*	609743	1q23.2	CADM3	Q8N126	1st- 3rd Decade
2.12	CMT type 2GG	606483	AD	*GBF1*	603698	10q24.32	GBF1	Q92538	2nd –3rd decade
2.13	CMT type 2HH	619574	AD	*JAG1*	601920	20p12.2	JAG1	P78504	1st- 3rd Decade
2.14	CMT type 2I	607677	AD	*MPZ*	159440	1q23.3	MPZ	P25189	4th-6th Decade
2.15	CMT type 2J	607736	AD	*MPZ*	159440	1q23.3	MPZ	P25189	4th – 6th decade
2.16	CMT type 2K	607831	AD/AR	*GDAP1*	606598	8q21.11	GDAP1	Q8TB36	< 3 Years
2.17	CMT type 2L	608673	AD	*HSPB8*	608014	12q24.23	HSPB8	Q9UJY1	2nd – 4th decade
2.18	CMT type 2M	606482	AD	*DNM2*	602378	19q13.2	*DNM2*	P50570	1st- 3rd Decade
2.19	CMT type 2N	613287	AD	*AARS*	601065	16q22.1	AARS	P49588	1st – 6th Decade

2.20	CMT type 2O	614228	AD	*DYNC1H1*	600112	14q32.31	DYNC1H 1	Q14204	1st Decade
2.21	CMT type 2P	614436	AR	*LRSAM1*	610933	9q33.3-q34.11	LRSAM1	Q6UWE0	1st – 8th decade
2.22	CMT type 2Q	615025	AD	*DHTKD1*	614984	10p14	DHTKD1	Q96HY7	2nd –3rd decade
2.23	CMT type 2R	615490	AR	*TRIM2*	614141	4q3.1	TRIM2	Q9C040	< 1 year
2.24	CMT type 2S	616155	AR	*IGHMBP2*	600502	11q13.3	IGHMBP2	P38935	1st Decade
2.25	CMT type 2T	617017	AD/AR	*MME*	120520	3q25.2	MME	P08473	4th – 6th Decade
2.26	CMT type 2U	616280	AD	*MARS*	156560	12q13.3	MARS	P56192	>50 years
2.27	CMT type 2V	616491	AD	*NAGLU*	609701	17q21.1	NAGLU	P54802	2nd – 7th Decade
2.28	CMT type 2W	616625	AD	*HARS*	142810	5q31.3	HARS	P12081	1st – 6th Decade
2.29	CMT type 2X	616668	AR	*SPG11*	610844	15q21.1	SPG11	Q96JI7	1st – 4th Decade
2.30	CMT type 2Y	616687	AD	*VCP*	601023	9p13.3	VCP	P55072	1st – 6th Decade
2.31	CMT type 2Z	616688	AD	*MORC2*	616661	22q12.2	MORC2	Q9Y6X9	1st-2nd decade
**Charcot-Marie-Tooth Disease Type 3 (CMT3) or Dejerine-sottas disease**									
3	CMT Type 3	145900	AD/AR	*MPZ*	601097	1q23.3	MPZ	P25189	1st-2nd decade
			AD/AR	*EGR2*	129010	10q21.3	EGR2	P11161	1st-2nd decade
			AD/AR	*PMP22*	159440	17p12	PMP22	Q01453	1st-2nd decade
			AD/AR	*PRX*	605725	19q13.2	PRX	Q9BXM0	1st-2nd decade
**Charcot-Marie-Tooth Disease Type 4 (CMT4)**									
4.1	CMT type 4A	214400	AR	*GDAP1*	606598	8q21.11	GDAP1	Q8TB36	< 2 years
4.2	CMT type 4B1	601382	AR	*MTMR2*	603557	11q21	MTMR2	Q13614	< 4 years
4.3	CMT type 4B2	604563	AR	*SBF2*	607697	11p15.4	SBF2	Q86WG5	4-13 years

4.4	CMT type 4B3	615284	AR	*SBF1*	603560	22q13.33	SBF1	O95248	5-20 years
4.5	CMT type 4C	601596	AR	*SH3TC2*	608206	5q32	SH3TC2	Q8TF17	1st – 4th Decade
4.6	CMT type 4D	601455	AR	*NDRG1*	605262	8q24.22	NDRG1	Q92597	5-25 years
4.7	CMT type 4E	605253	AD/AR	*EGR2/MPZ*	129010	10q21.3	EGR2	P11161	Congenital
4.8	CMT type 4F	614895	AR	*PRX*	605725	19q13.2	PRX	Q9BXM0	< 1 years
4.9	CMT type 4G	6052850	AR	*HK-1*	142600	10q22.1	HK-1	P19367	8-16 years
4.10	CMT type 4H	609311	AR	*FGD4*	611104	12p11.21	FGD4	Q96M96	< 2 years
4.11	CMT type 4J	611228	AR	*FIG4*	609390	6p21	FIG4	Q92562	1st – 6th Decade
4.12	CMT type 4K	616684	AR	*SURF1*	185620	9p34.2	SURF1	Q15526	1st decade
**Charcot-Marie-Tooth Disease X-linked Type (CMTX)**									
5.1	CMT X1	302800	XL-D	*GJB1*	304040	Xq13.1	GJB1	P08034	1st-2nd decade
5.2	CMT X2	302801	XL-R	*?*	?	Xp22.2	?	?	< 1 years
5.3	CMT X3	301802	XL-R	*?*	?	Xq26	?	?	10-14 years
5.4	CMT X4	310490	XL-R	*AIFM1*	300169	Xq26.1	AIFM1	O95831	< 4 years
5.5	CMT X5	311070	XL-R	*PRPS1*	311850	Xq22.3	PRPS1	P60891	1st decade
5.6	CMT X6	300905	XL-D	*PDK3*	300906	Xp22.11	PDK3	Q15120	1st decade
**Dominant Intermediate Charcot-Marie-Tooth Disease (DI-CMT)**									
6.1	CMT-DI A	606483	AD	*GBF1*	603698	10q24.32	GBF1	Q92538	2nd Decade
6.2	CMT-DI B	606482	AD	*DNM2*	602378	19q13.2	DNM2	P50570	1st-2nd decade
6.3	CMT-DI C	608323	AD	*YARS*	603623	1p35.1	YARS	P54577	1st – 6th Decade
6.4	CMT-DI D	607791	AD	*MPZ*	601097	1q23.3	MPZ	P25189	---

6.5	CMT-DI E	614455	AD	*INF2*	610982	14q32.33	INF2	Q27J81	1st-3rd decade
6.6	CMT-DI F	615185	AD	*GNB2*	139390	7q22.1	GNB2	P63244	1st-2nd decade
6.7	CMT-DI G	617882	AD	*NEFL*	162280	8p21.1	NEFL	P07196	1st-2nd decade
**Recessive Intermediate Charcot-Marie-Tooth Disease (RI-CMT)**									
6.8	CMT-RI A	608340	AR	*GDAP1*	606598	8q21.11	GDAP1	Q8TB36	2-4 years
6.9	CMT-RI B	613641	AR	*KARS*	601421	16q23.1	KARS	Q15046	---
6.10	CMT-RI C	615376	AR	*PLEKHG5*	611101	1p36.31	PLEKHG 5	O94827	1st – 5th Decade
6.11	CMT-RI D	616039	AR	*COX6A1*	602072	12q24.31	COX6A1	P12074	4-5 years

Table S2: *In-silico* predictions of the identified variants in their respective genes by online prediction tools

		***MPV17*: c.83G>**		***PRX*: c.231C>A**		***GJB1* c.61G>C**		***SACS*: c.262C>T**		***SACS*: c.4934G>C**	
**S/No**	**Engine**	**Score**	**Indicative Prediction**	**Score**	**Indicative Prediction**	**Score**	**Indicative Prediction**	**Score**	**Indicative Prediction**	**Score**	**Indicative Prediction**
**1**	BayesDel addAF	0.4419	Damaging	0.618	Damaging	0.7408	Damaging	0.625	Damaging	0.5022	Damaging
**2**	BayesDel noAF	0.3971	Damaging	0.6499	Damaging	0.8263	Damaging	0.66	Damaging	0.4836	Damaging
**3**	MetaLR	0.7972	Damaging	n/p, n/a	n/p, n/a	0.9901	Damaging	n/p, n/a	n/p, n/a	0.8923	Damaging

**4**	MetaRNN	0.9275	Damaging	n/p, n/a	n/p, n/a	0.9942	Damaging	n/p, n/a	n/p, n/a	0.9386	Damaging
**5**	MetaSVM	0.5284	Damaging	n/p, n/a	n/p, n/a	0.9692	Damaging	n/p, n/a	n/p, n/a	1.0003	Damaging
**6**	REVEL	0.787	n/p, n/a	n/p, n/a	n/p, n/a	0.971	n/p, n/a	n/p, n/a	n/p, n/a	0.8489	n/p, n/a
**7**	BLOSUM	8	n/p, n/a	n/p, n/a	n/p, n/a	6	n/p, n/a	n/p, n/a	n/p, n/a	-5	n/p, n/a
**8**	DANN	0.9962	n/p, n/a	0.9934	n/p, n/a	0.999	n/p, n/a	0.9971	n/p, n/a	0.9913	n/p, n/a
**9**	DEOGEN2	0.6296	Damaging	n/p, n/a	n/p, n/a	0.8818	Damaging	n/p, n/a	n/p, n/a	0.7455	Damaging
**10**	EIGEN	0.3549	n/p, n/a	0.2456	n/p, n/a	n/p, n/a	n/p, n/a	0.6421	n/p, n/a	0.5472	n/p, n/a
**11**	EIGEN PC	0.3916	n/p, n/a	0.002112	n/p, n/a	n/p, n/a	n/p, n/a	0.4614	n/p, n/a	0.4478	n/p, n/a
**12**	FATHMM	2.11	Damaging	0.4617	Neutral	6.3	Damaging	n/p, n/a	n/p, n/a	-2.99	Damaging
**13**	FATHMM-MKL	0.9467	Damaging	0.1743	Neutral	0.9715	Damaging	0.9542	Damaging	0.9821	Damaging
**14**	FATHMM-XF	0.7566	Damaging	n/p, n/a	n/p, n/a	n/p, n/a	n/p, n/a	0.7482	Damaging	0.9591	Damaging
**15**	LIST-S2	0.8637	Damaging	n/p, n/a	n/p, n/a	0.893	Damaging	n/p, n/a	n/p, n/a	0.9291	Damaging
**16**	LRT	-0.0	Deleterious	0	Deleterious	0	Deleterious	0.000006	Deleterious	0	Deleterious
**17**	M-CAP	0.5715	Damaging	n/p, n/a	n/p, n/a	0.9597	Damaging	n/p, n/a	n/p, n/a	0.8277	Damaging

**18**	Mutation assessor	2.48	Medium	n/p, n/a	n/p, n/a	4.015	High	n/p, n/a	n/p, n/a	3.355	Medium
**19**	MutationTaster	1, 0.9999	Disease causing	1	Disease causing	1	Disease causing	1	Disease causing	0.9993	Disease causing
**20**	MVP	0.9123	n/p, n/a	n/p, n/a	n/p, n/a	0.9998	n/p, n/a	n/p, n/a	n/p, n/a	0.9294	n/p, n/a
**21**	PrimateAI	0.7167	Tolerated	n/p, n/a	n/p, n/a	0.9061	Damaging	n/p, n/a	n/p, n/a	0.7239	Tolerated
**22**	PROVEAN	4.44	Damaging	n/p, n/a	n/p, n/a	7.22	Damaging	n/p, n/a	n/p, n/a	-6.28, -6.04	Damaging
**23**	SIFT	-0.045	Damaging	n/p, n/a	n/p, n/a	0	Damaging	n/p, n/a	n/p, n/a	0	Damaging
**24**	SIFT4G	-0.028	Damaging	n/p, n/a	n/p, n/a	-0.001	Damaging	n/p, n/a	n/p, n/a	0	Damaging

n/p: not predicted by online in-silico tools, n/a: not available
